# Gut microbiome of century-old snail specimens stable across time in preservation

**DOI:** 10.1186/s40168-022-01286-z

**Published:** 2022-06-29

**Authors:** Bridget N. Chalifour, Leanne E. Elder, Jingchun Li

**Affiliations:** 1 Department of Ecology and Evolutionary Biology, University of Colorado Boulder, 1900 Pleasant Street, 334 UCB, Boulder, CO 80309 USA; 2 Museum of Natural History, University of Colorado Boulder, 265 UCB, Boulder, CO 80309 USA; 3 New Zealand Arthropod Collection, Manaaki Whenua Landcare Research, 231 Morrin Road St. Johns, Auckland, NZ 1072 New Zealand

**Keywords:** Gut microbiome, Mollusk, Natural History Museum, Gastropod, 16S rRNA gene, Invertebrates

## Abstract

**Background:**

Museum biological specimens provide a unique means of gathering ecological information that spans wide temporal ranges. Museum specimens can also provide information on the microbial communities that persist within the host specimen. Together, these provide researchers valuable opportunities to study long-term trends and mechanisms of microbial community change. The effects of decades-long museum preservation on host-microbial communities have not been systematically assessed. The University of Colorado’s Museum of Natural History has densely sampled Oreohelix strigosa (Rocky Mountainsnail) for the past century; many are preserved in ethanol, which provides an excellent opportunity to explore how the microbiome changes across time in preservation.

**Results:**

We used 16S rRNA (ribosomal ribonucleic acid) gene amplicon sequencing to examine *Oreohelix strigosa* gut microbiomes from museum specimens across a 98-year range, as well as within short-term preservation treatments collected in 2018. Treatment groups included samples extracted fresh, without preservation; samples starved prior to extraction; and samples preserved for 1 month, 6 months, and 9 months. General microbiome composition was similar across all years. Sample groups belonging to specific years, or specific short-term treatments, showed unique associations with select bacterial taxa. Collection year was not a significant predictor of microbial richness, though unpreserved short-term treatments showed significantly higher richness than preserved treatments. While the year was a significant factor in microbiome composition, it did not explain much of the variation across samples. The location was a significant driver of community composition and explained more of the variability.

**Conclusions:**

This study is the first to examine animal host-associated microbiome change across a period of nearly one century. Generally, geographic location was a greater factor in shaping gut microbiome composition, rather than a year collected. Consistent patterns across this temporal range indicate that historic specimens can answer many ecological questions surrounding the host-associated microbiome.

Video Abstract

**Supplementary Information:**

The online version contains supplementary material available at 10.1186/s40168-022-01286-z.

## Introduction

The microbiome is an evolutionary adaptation that has allowed many animals to capture and utilize resources in their environment [1]. The gut microbiome is particularly important to study as it provides insight into both host-specific and population-level subtle changes in environmental patterns and microbial communities. It is imperative to understand how microbiomes shift over broad temporal and spatial gradients as changes in the microbiome can impact the host response to stressors [2]. Analyzing past trends in microbiome shifts is also paramount to predicting future trends and safeguarding rare species that depend on their symbiotic microbes [3]. Historic museum specimens provide the long-term data needed to form a more comprehensive picture of how environmental changes impact microbial communities and predict how microbiomes will shift due to future changes [2, 4]. The specimens also provide a unique and often underappreciated means of gathering ecological information spanning wide geographic ranges [5].

So far, studies using museum collections have mainly focused on the host, and not organisms living on or within the host [6], especially not microbiomes associated with the specimens. Microbiomes associated with other biological samples can now be routinely characterized, thanks to the development of high-throughput sequencing approaches, which are cost-effective and able to characterize previously difficult to culture bacteria [7]. This development has widened the possible scale and narrowed the effort needed to describe the microbiomes of museum specimens, allowing microbiome researchers to conduct expansive studies with limited field work.

While microbiome compositions in animal hosts are not significantly altered by short-term preservation [8], the effects of decades-long preservation require a further study. As far as we know, no studies have directly tested how different time periods in ethanol preservative impact animal-associated microbial abundance, diversity, and community structure over a decades-long time scale. To address this issue, we need a model species with an extensive presence and wide temporal range in museum collections and known to harbor a core gut microbiome.

Land snails are dispersal-limited, due to the high energy cost of movement [9]. As low-vagility organisms, many land snails must survive within a limited range. If a habitat does not meet certain qualifications, they must adapt or perish. The gastropod microbiome is theorized to influence the host’s physiological state, confer protection against pathogens, and regulate host immune function, along with enabling host dietary diversity [10, 11]. Therefore, the snail microbiome may play an important role in the host’s adaptation, tolerance to environmental perturbations, and immune system functioning [7, 12]. *Oreohelix strigosa*, more commonly known as the Rocky Mountainsnail, is a pulmonate terrestrial gastropod belonging to the class Gastropoda (Gould, 1846, Fig. 1A, B). *O. strigosa* is widespread across the mountainous western USA, found in a multitude of habitats, ranging from grassy fields to the talus slopes of the Rocky Mountains. More importantly, the University of Colorado Museum of Natural History (CUMNH) has densely sampled *O. strigosa* for the past century due to its ubiquitous presence in Colorado, which has interested past curators and students in collection and research opportunities using these easily accessible snails. Many of these snails are preserved in ethanol and therefore appropriate for molecular research. The CUMNH wet collection contains thousands of preserved specimens of *O. strigosa*, ranging in collection date from 1915 to 2019. Previous research has indicated the presence of a stable gut microbiome in *O. strigosa* specimens [13], making it an ideal system to compare microbiome quality through time.

**Fig. 1:**
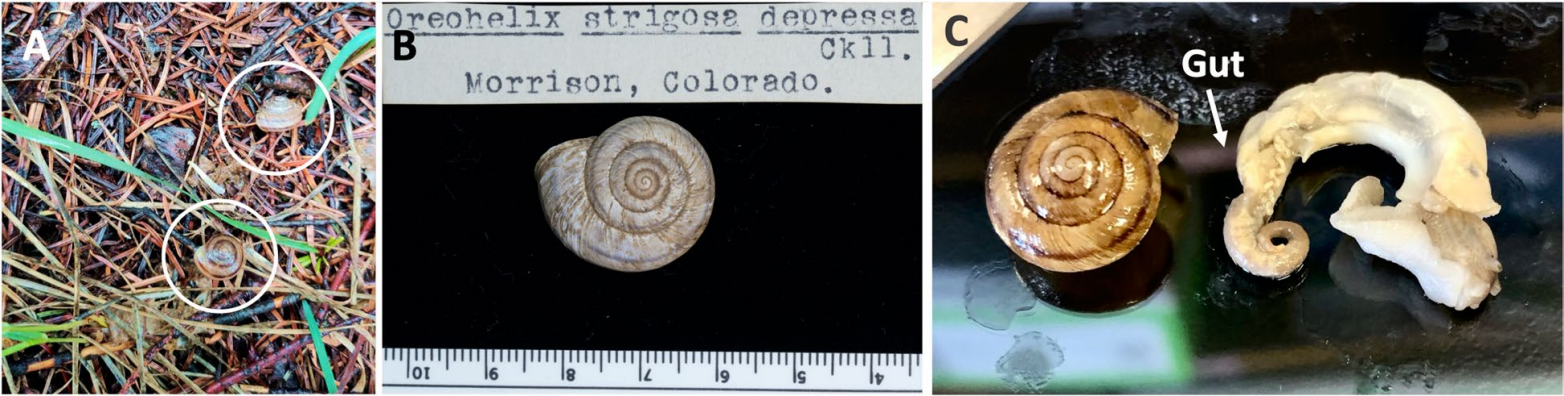
**A** Two adult *Oreohelix strigosa* individuals (circled) grazing in leaf litter, **B** a dry *O. strigosa* specimen from the University of Colorado Museum of Natural History (University of Colorado Museum (UCM) Catalog Number 2928, originally collected from Morrison, Colorado, USA, in September 1911), and **C** a dissected and complete *O. strigosa* internal body with arrow pointing to where the gut tissue was extracted, alongside shell. Photo A by J. Li, photos B and C by B. Chalifour

The aims of this study were threefold: (1) to test the applicability of high-throughput sequencing techniques on describing animal gut microbiomes using museum specimens, (2) to compare historic museum specimen microbiomes to contemporary samples, and (3) to explore potential driving factors of microbiome variability in museum samples. We investigated the impacts of preservation in both long-term (15–98 years) and short-term (0–9 months) ethanol-preserved samples. We examined taxonomic changes, changes in microbial composition, as well as changes in microbial richness, or number of operational taxonomic units (OTUs) across snails’ gut microbiomes.

Though there is a substantial dearth of research examining the long-term effects of museum preservation on the microbiome, one study has shown that museum-preserved fish microbiomes can be captured through high-throughput sequencing, though this study had small sample sizes likely due to formalin-fixated samples which contributed to high dropout rates in the data [6]. Another study by Neu et al. (2021) showed that bivalve tissue preserved in ethanol for up to 10 years was successful at providing high-resolution information regarding trends in microbial community compositions [2]. Other studies have found that microbial richness is less dramatically affected by most preservation methodologies in short-term storage experiments [14] and that methodological consistency is more important than time of preservation [15]. Based on these past findings, we predicted that microbial community composition and diversity would not significantly differ among sample populations from different years. Collectively, this work advances our knowledge of the applicability of decades-old museum specimens to high-throughput sequencing projects and broadens knowledge on terrestrial gastropod gut microbiomes.

## Materials and methods

This study consists of data from 112 snails from two sources: 55 historical collected specimens from the University of Colorado Museum of Natural History’s (CUMNH) Invertebrate Zoology collections (for specimen information, see Supplementary Table 1) and 57 field collected specimens from localities within the state of Colorado (deposited in the CUMNH Invertebrate Zoology collections, see Supplementary Table 1).

### Museum sampling

The 55 historical specimens from the CUMNH collections were collected between 1920 and 2004. The sample selection from museum collections was based on (1) the sample size within each jar—in order to leave intact specimens for future study and morphological reference, we only sampled from lots that contained at least 15 specimens; (2) the distance between time points, as we strived to achieve both even spacing between years and a wide temporal spread of years in preservation. Based on these criteria, 3 samples were taken from the year 1920, 15 from 1974, 2 from 1980, 13 from 1982, 2 from 2000, and 20 from 2004 (Table 1).

**Table 1:** Metadata from all snail samples

	**GPS coordinates (estimated pre-2018)**	**Year collected**	**Palmer drought index**	**Estimated elevation (in ft):**	**Average microbial richness ± standard deviation**	**Total snails**
Mountain Research Station (MRS)	40.0314420, − 105.5394289	2018	Severe drought	9675	483.36 ± 386.15	15
Vail	39.6449151, − 106.3146577	2018	Extreme drought	8353	492.84 ± 174.17	34
Frisco	39.5752723, − 106.1181343	2018	Extreme drought	9298	425.36 ± 277.72	8
Steamboat Springs (2004)	40.4817935, − 106.8245969	2004	Severe drought	6505	315.91 ± 193.95	13
Glen Eden	40.748779, − 106.843133	2004	Severe drought	7513	288.29 ± 64.13	7
Silverthorne	39.8356, − 106.31891	2003	Mid-range	8586	493.33 ± 376.47	3
Pagoda	40.321214, − 107.344139	2000	Severe drought	7008	122.50 ± 15.56	2
Redstone	39.199847, − 107.232233	1982	Very moist	7159	379.89 ± 119.12	9
Telluride	37.934889, − 107.799037	1982	Very moist	8809	459.25 ± 286.37	4
Steamboat Springs (1980)	40.472242, − 106.873814	1980	Mid-range	8192	231.50 ± 19.09	2
Rifle	39.727398, − 107.68823	1974	Moderate drought	9583	592.50 ± 422.56	4
Glenwood Springs	39.577612, − 107.369814	1974	Moderate drought	6141	496.18 ± 158.64	11
Boulder	39.929963, − 105.293328	1920	Moderately moist	6237	245.33 ± 152.53	3

### Field sampling

**Fig. 2:**
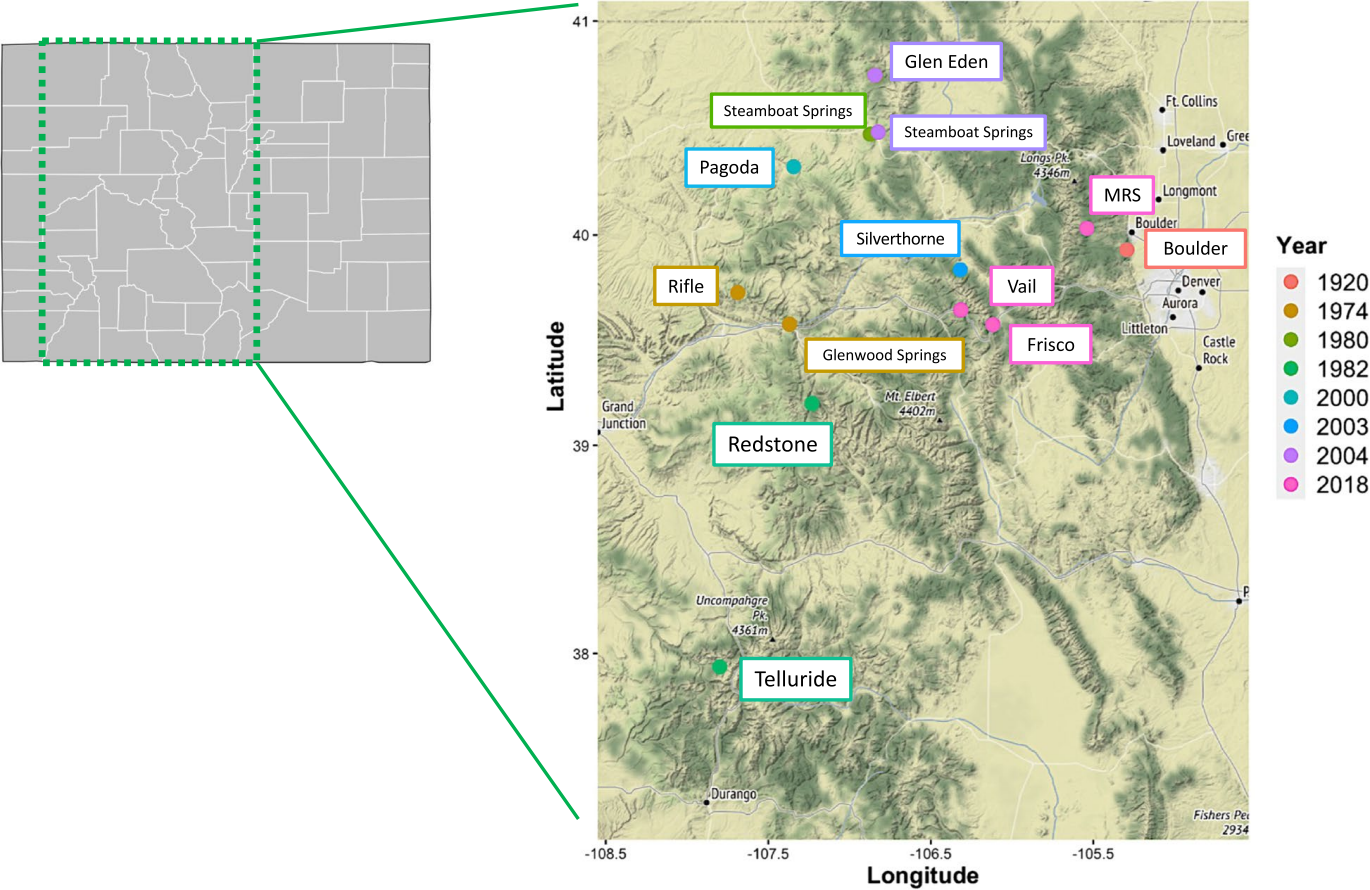
Map of the front range region of the state of Colorado, with collection points and their corresponding years indicated

In the summer months of 2018 (between July and September) when terrestrial snails of the Rockies are most active, we collected fresh, living samples of *Oreohelix strigosa* from three locations within the Colorado Front Range: (1) the University of Colorado Mountain Research Station in Ward, Colorado (MRS); (2) the 10-mile trail in Frisco, Colorado; and (3) the Gore Valley trail in Vail, Colorado. These freshly collected samples from 2018 (*N* = 57) included 15 from the MRS, 8 from Frisco, and 34 from Vail (Table 1). We used a qualitative collection method, i.e., collections made by direct visual searching, to collect specimens for this study, in accordance with Chalifour & Li [13] and Coppolino [16]. All collections were taken with the appropriate permitting for invertebrates. See Fig. 2 for a complete map of collection localities and corresponding years.

Live snails were first drowned in distilled water and preserved in 95% ethanol for 24 h, then transferred to and kept in 80% ethanol for permanent preservation as they were extracted, in accordance with CUMNH policies. This methodology has worked well on *O. strigosa* to increase plasticity in the tissue and ease of removal of the whole body from the shell for precise gut dissections [13]. This is also consistent with the methodology used by some previous researchers who have deposited *O. strigosa* specimens in to the CUMNH collections, according to CUMNH records.

Samples classified as “fresh” underwent no preservation and were extracted immediately after sacrifice. For a subset of each locality population, we extracted gut tissue periodically at ethanol preservation lengths of 1, 6, and 9 months. This resulted in 11 fresh samples, 10 1-month preserved samples, 14 6-month preserved samples, and 17 9-month preserved samples (Table 2). Additionally, snails from each 2018 locality (a total of six samples) were kept under starvation conditions directly after collection with a natural photoperiod for approximately 1 week. They were given no food supplement of any kind. These starved snails were then sacrificed in the same manner as the other live snails.

**Table 2:** Breakdown of short-term treatment sample sizes within 2018 snails

Location	Fresh	1 Month	6 Months	9 Months	Starved
Mountain Research Station	2	3	4	5	1
Vail	6	6	9	10	3
Frisco	2	1	1	2	2
**Total**	**11**	**10**	**14**	**17**	**6**

### Microbial DNA extraction

All dissections in this study were performed aseptically, using sterile instruments. The soft body of the snail was removed by using fine forceps to gently pull the entire body out by the foot. In some cases, particularly of the older specimens, the soft body tissue had become too tough and could not be pulled out by the foot, so the apex of the shells was carefully broken and removed to expose the soft body, and then, the whole soft body was removed from the shells using forceps. The digestive tract was carefully isolated from the body, and a portion of gut was collected (Fig. 1C). We then re-preserved all shell and body parts individually in 80% ethanol after the necessary tissue was removed and re-catalogued these in the CUMNH collection accordingly.

We extracted genomic DNA from the snail gut tissue using the E.Z.N.A. Mollusk DNA Extraction kit according to the manufacturer’s instructions (Omega BioTek, Norcross, GA, USA), as shown by our preliminary trials to be the optimal kit for microbial extraction of mollusk tissue by measures of DNA concentration. The V4 hypervariable region of the 16S rRNA gene was amplified by PCR with the 515F/806R primer pair modified to include Illumina adapters and appropriate error-correcting barcodes, as comparable with other microbiome studies [17]. PCR amplification protocol was taken from the Earth Microbiome Project protocol for 515F/806R [17]. Library preparation and sequencing were facilitated by the Center for Microbial Exploration at the University of Colorado Boulder. 150 bp single-indexed paired-end reads were generated on an Illumina Miseq platform PE300 (Illumina Corporation, San Diego, CA, USA) using a 2-by-150-bp paired end chemistry with the MiSeq V2 300-cycle kit (Illumina, San Diego, CA, USA). Samples were sequenced on one Illumina MiSeq run at the University of Colorado Next-Generation Sequencing Facility through BioFrontiers (Boulder, CO, USA).

Data were processed using the USEARCH10 pipeline [18] and the same processing as used in Chalifour & Li [13]. Reads were merged with a minimum overlap of 16 bp (usearch8 -fastq_mergepairs). Trimmed reads were quality-filtered with a max error rate of 1.0 (usearch10 -fastq_filter; 96.2% passed). Unique sequences were identified using usearch10 -fastx_uniques which clustered as 99% 16S rRNA gene operational taxonomic units (OTUs) with usearch10 -cluster_otus uniques.fa. OTUs were classified taxonomically using the GreenGenes 13_8 database [19]. We removed OTUs that were classified as mitochondria or chloroplasts, and we did not use any samples that yielded fewer than 2000 reads per sample. Samples were not rarefied after exclusion. Six of the 112 snail gut samples (four 2018 samples, two 2004 samples) failed to meet this threshold for sequencing depth and were excluded from downstream analyses, as were extraction and PCR blanks. Available data may be found at FigShare using (links to be added upon acceptance).

### Statistical analyses

Data analysis was completed with R statistical software [20]. In sum, we examined community composition differences between our major treatment groups (i.e., explanatory variables), including the year collected, short-term preservation treatments, and location, as well as how several other ecological factors impact the community compositions. Once we determined how different the community compositions were, we identified which specific microbial taxa were driving the differences, using indicator species analyses. We then examined how microbial richness was affected by several explanatory variables, including year, short-term preservation treatment, and location. We also investigated species evenness and Shannon index as factors of microbial richness.

To evaluate and visualize the taxonomic makeup of our treatment groups, we ran Kruskal–Wallis tests comparing relative abundances of bacterial families across both years collected and short-term preservation treatments using the “taxa_summary_by_sample_type” function in {MCToolsR} and visualized the taxonomic compositions with the “plot_taxa_bars” function in {MCToolsR} [21].

Snail gut microbial compositional differences were assessed using a non-metric multidimensional scaling analysis (NMDS) based on year preserved, the short-term treatments of 2018 samples, and location. We used a Bray–Curtis distance matrix with a multiple regression analysis of all snails’ gut microbiomes using microbial community diversity as the dependent variable for each explanatory variable (year, short-term preservation treatment, location). The NMDS allows us to visualize differences between gut community compositions based on groupings but does not give an indication of significant differences. Therefore, we subsequently used a permutation analysis of variance (PERMANOVA), a nonparametric test similar to an analysis of variance (ANOVA), to test for significant differences in microbial compositions among different treatment groups (“adonis2” function in {vegan} package) [22].

As part of the PERMANOVAs, we also explored how environmental factors that may impact gastropod physiology affect the gut microbiome compositions. The environmental factors included Palmer drought index (a measure of dryness based on precipitation and temperature) and elevation. We used the historic Palmer drought indices maps from the National Oceanic and Atmospheric Administration (NOAA) [23] to find the Palmer drought index for the month and year of the geographic region where each sample was collected. We used The National Map from the United States Geological Survey (USGS) [24] to estimate elevation for the location collected based on estimated latitude and longitude.

As geographic distances can be a major factor in shaping the microbial compositions of other animal microbiomes, we also ran a Mantel test to examine if there was any correlation between snail population geographic distance and microbial community similarity. The test was run in the {vegan} and {geosphere} R packages and tested for correlation between a geographic distance matrix of the Haversine distances of our estimated latitudes and longitudes and bacterial species abundance Bray–Curtis dissimilarity matrix [22, 25]. We used the {ggmap} R package [26] to plot collection points in Fig. 2.

After determining where microbial compositions across these treatment groups significantly differed, we identified which specific bacterial species were making up the core microbiome across all samples and driving differences between groups. We used the “return_top_taxa” function of the {MCToolsR} package to initially discern which taxa were most prevalent across all snail gut samples and give insight into the core microbiome [21]. To determine which bacterial species were associated with our different treatment groups, we used a similarity percentage (SIMPER) test using the “simper” function of the {vegan} package [27]. This test performs pairwise comparisons of treatment groups and finds the contribution of each bacterial species to the average between-group Bray–Curtis dissimilarity. We followed the SIMPER with a multilevel analysis of pattern (multipatt) using the “multpatt” function of the {indicspecies} package [28]. The mulitpatt shows bacterial species that are significantly associated to treatment groups or treatment group combinations.

We tested not only how microbiome composition changed across these explanatory variables, but also how microbial richness changed. We used linear models to determine if year and the short-term treatment groups were significant predictors of microbial richness. As the response variable of microbial richness was non-normally distributed, and the relationship between microbial richness and collection year was non-linear, we used a negative binomial generalized linear model (see equations below) to test both the long-term preservation data (Eq. 1) and the 2018 short-term preservation data (Eq. 2). As there were no samples from every Palmer drought index taken at every time point, we could not determine how OTU richness was expected to change for a given index across the 98-year time range, so we used a separate negative binomial generalized linear model to test the indices from the long-term data (Eq. 3). Negative binomial generalized linear models using the R package {MASS} were used to model microbial richness as a function of Palmer drought index and of the fixed covariates: year, location, and elevation, and short-term preservation treatment and location. We also tested log-transformed and Poisson distribution generalized linear models along with the negative binomial generalized linear model against a null model to determine which model had the best fit. A Poisson distribution did not describe this data well because although microbial richness was still discrete, count data, the data was over-dispersed as the variance greatly exceeded the mean. The AIC (Akaike information criterion) score of the negative binomial model was lowest and therefore was the test chosen. Relevant predictors and their corresponding coefficient values and *p*-values are reported in Tables 3 and 4.

**Table 3:** Negative binomial generalized linear model predictors, coefficients, and *p*-values for models containing samples across all years

Predictor	Coefficient	*P*-value
Year	0.015	0.353
Location—Frisco	− 0.967	0.559
Location—Glen Eden	− 1.125	0.432
Location—Glenwood Springs	− 0.124	0.896
Location—Mountain Research Station (MRS)	− 0.822	0.619
Location—Pagoda	− 1.888	0.178
Location—Redstone	− 0.501	0.642
Location—Rifle	0.043	0.965
Location—Silverthorne	-0.577	0.687
Location—Steamboat Springs	-0.981	0.470
Location—Telluride	-0.331	0.762
Location—Vail	-0.803	0.626
Elevation	< 0.001	0.229

Boulder was used as the model intercept location

**Table 4:** Negative binomial generalized linear model predictors, coefficients, and *p*-values for model containing only 2018 samples

Predictor	Coefficient	*P*-value
Short-term treatment—6 months	0.328	0.109
Short-term treatment—9 months	-0.113	0.573
Short-term treatment—fresh	0.515	0.030 *
Short-term treatment—starved	0.638	0.014 *
Location—Mountain Research Station (MRS)	0.127	0.577
Location—vail	0.205	0.307

^*^Significant values. Frisco, 1-month preservation was the model intercept

1 $$\text{Long term richness}=\alpha+\upbeta_1\left(\text{year collected}\right)+\upbeta_2\left(\text{location}\right)+\upbeta_3\left(\text{elevation}\right)+\text{error}$$

2 $$\text{Short term richness}=\alpha+\upbeta_1(\text{short term treatment})+\upbeta_2(\text{location})+\text{error}$$

3 $$\text{Richness}=\alpha+\upbeta_1(\text{Palmer drought index})+\text{error}$$

Along with microbial richness, we also looked at evenness and Shannon index. We used Kruskal–Wallis tests to examine how evenness and Shannon indices changed based on year and short-term preservation treatment.

## Results

### Taxonomic composition of gut bacterial community

The microbiome composition of *Oreohelix strigosa* historical museum samples proved to be highly diverse. In total, there were 5,233,457 reads across all non-filtered out samples. The average number of reads per snail gut was 42,548.43 ± standard deviation (SD) 19,689.78, with a maximum number of reads of 81,789 and a minimum number of 2948 reads. The identified OTUs belonged to 37 unique phyla, 233 families, and 446 genera.

Three OTUs were found in 100% of gut samples and accounted for 1,534,035 reads, or roughly 32% of all reads. These were OTU_10 and OTU_9 (both members of bacterial family Enterobacteriaceae) and OTU_17 (*Sphingomonas* sp.). These three core taxa were also found in the top taxa across all samples. In 90% of gut samples, 22 OTUs accounted for 2,629,397 (55%) reads. In 80% of gut samples, 39 OTUs accounted for 2,843,219 (59%) reads. Other top taxa included *Sphingobacterium faecium*, *Serratia* sp., *Lactococcus* sp., *Rahnella aquatilis*, *Spirosoma* sp., *Yersinia* sp., *Enterococcus* sp., *Spingomonas* sp., and other Enterobacteriaceae members.

In total, historic samples and 2018 samples shared 3603 OTUs. There were 1852 OTUs unique to historic samples, and 1356 OTUs unique to 2018 samples. These common OTUs largely came from phylum Proteobacteria, which accounted for roughly 41% of OTUs, followed by Bacteroidetes (16%) and Actinobacteria (14%). At the family level, the family with the most OTUs that was identifiable was Chinophagaceae, followed by Sphingomonadaceae and Chthoniobacteraceae.

**Fig. 3:**
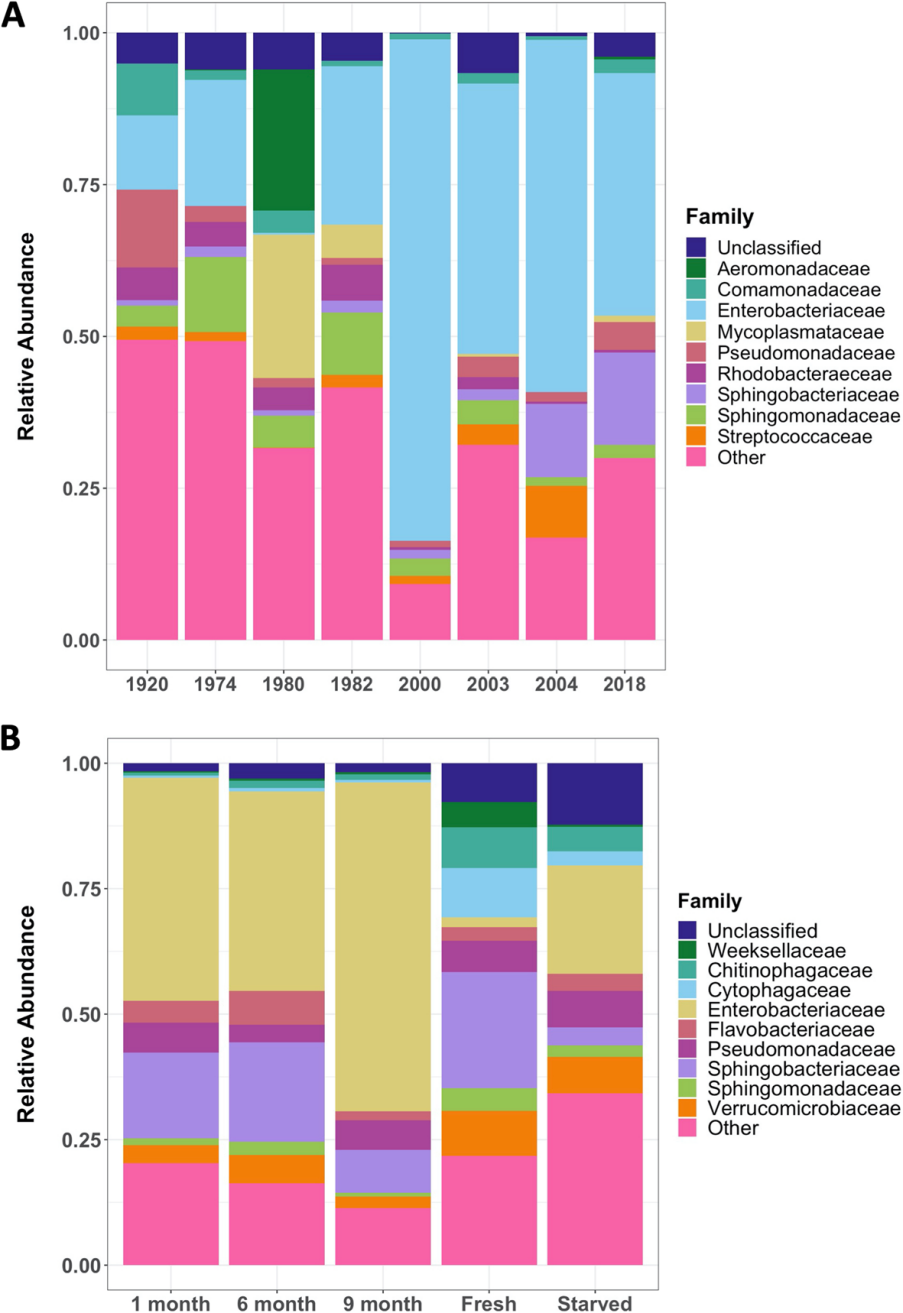
**A** Relative abundance of the top ten bacterial families contributing to each snail sample set collected from the years 1920–2018 and **B** relative abundance of the top ten bacterial families contributing to each snail sample set from 2018. “Other” refers to the cumulative abundance of all other families not included in the top ten

At the family level, most collection years did not show significant differences of bacterial relative abundances (Fig. 3A). Kruskal–Wallis tests showed Enterobacteriaceae abundance was much lower in 1980 samples than other years at 0.3%, and much higher in 2000 samples at 82%. Samples from 2004 showed significantly higher abundances of Streptococcaeceae and Sphingobacteriaceae.

Short-term treatments also showed significant differences between relative abundances of bacterial families (Fig. 3B). Snails from the fresh treatment had significantly lower levels of Enterobacteriaceae and higher levels of Chitinophagaeceae than the other treatments. Starved snails had significantly lower levels of Sphingobacteriaceae than the other treatments.

### Microbial composition

**Fig. 4:**
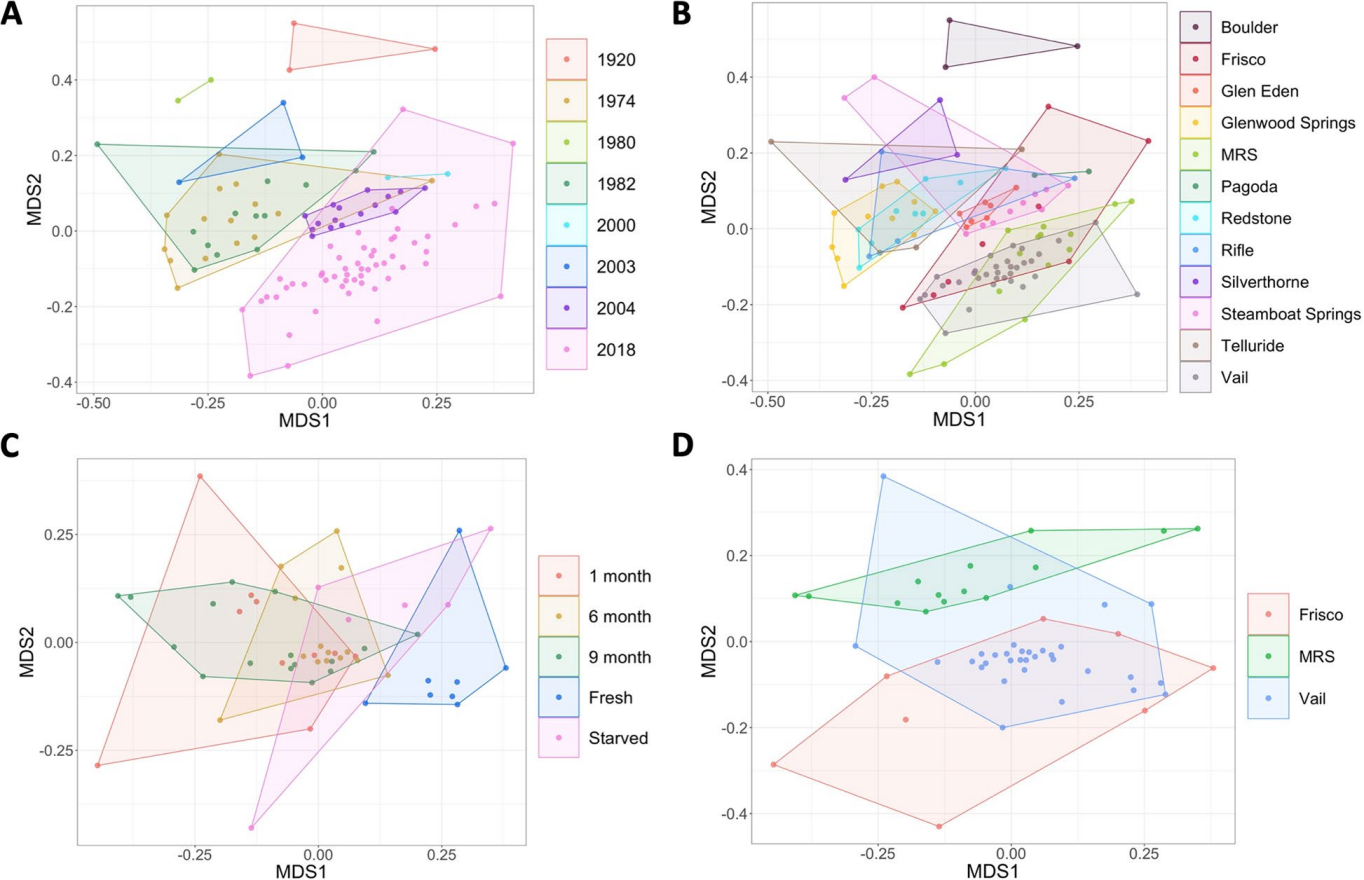
Non-metric multidimensional scaling analysis based on **A** year collected, for all snails from 1920 to 2018 (PERMANOVA: *p*-value < 0.001, *R*^2^ = 0.02), **B** location collected, for all snails from 1920 to 2018 (PERMANOVA: *p*-value < 0.001, *R*^2^ = 0.12), **C** short-term preservation treatment, for all snails from 2018 (PERMANOVA: *p*-value < 0.001, *R*^2^ = 0.20), and **D** location collected, for all snails from 2018 (PERMANOVA: *p*-value < 0.001, *R*.^2^ = 0.18). Each point represents one snail gut microbiome community. MRS refers to the Mountain Research Station location

Across the entire dataset, the location explained more variation in microbial communities than year. When controlling for location, the year *O. strigosa* samples were collected was a significant predictor of microbiome composition, but only explained 2.25% of variability between samples (Fig. 4A). In contrast, when controlling for year, the location was again a significant predictor and explained 12.38% of variability between samples (Fig. 4B).

Within the 2018 dataset, short-term preservation treatment (including fresh, starved, 1-month, 6-month, and 9-month treatments separately) controlling for the location was a significant predictor and explained 19.56% of variation (Fig. 4C). The location was also a significant predictor of microbiome composition and explained 16.86% of the variation between microbial community compositions when controlling for short-term preservation treatment (Fig. 4D). More specifically, when categorizing short preservation treatments binarily as either preserved or not preserved, the presence of preservation explained much less (9.59%) of the variation as location (17.15%).

We also investigated other parameters that might explain some of the variation across gut community compositions. When controlling for year, the Palmer drought index was a significant predictor of microbiome composition, but only explained 4.60% of the variation between sample compositions. When controlling for location, the Palmer drought index was a significant predictor of microbiome composition, but only explained 2.23% of the variation between sample compositions. Elevation of the collection site similarly was a significant predictor that explained very little of the variation across communities, only explaining 2.29% when controlling for year, and 2.64% when controlling for location.

A Mantel test comparing geographic distance and species abundance dissimilarity showed that the physical distances between all samples was significantly correlated with Bray–Curtis dissimilarity (Mantel statistic *R* = 0.24, *p*-value < 0.001). As samples became more dissimilar in geographic location, they also became more dissimilar in microbial community composition. This result holds true when analyzing microbial community composition from only 2018 samples, again, the geographic distance matrix has a moderate, but highly significant relationship with the species Bray–Curtis dissimilarity matrix (Mantel statistic *R* = 0.27, *p*-value < 0.001).

### Indicator species analysis

The top five taxa that contributed to differences between snails collected in 1920 and snails collected in 2018 were OTU_9 and OTU_10 (both members of family Enterobacteriaceae), OTU_1 (*Sphingobacterium faecium*), OTU_27 (*Pseudomonas* sp*.*), and OTU_64 (*Delftia* sp*.*). The Enterobacteriaceae members and *S. faecium* dominated the 2018 samples over the 1920 samples, while the opposite was true for the *Pseudomonas* sp*.* and *Delftia* sp*.*

The top five taxa that contributed to differences between snails that underwent 9 months of preservation and fresh snails were OTU_9 and OTU_10 (both members of family Enterobacteriaceae), OTU_13 (a member of family Sphingobacteriaceae), OTU_1 (*Sphingobacterium faecium*), and OTU_18 (*Spirosoma* sp*.).* The Enterobacteriaceae members and *S. faecium* were more common in the 9-month samples than the fresh samples, while the opposite was true for the Spingobacteriaceae and *Spirosoma* sp. When simply looking at preserved versus unpreserved specimens from 2018, four out of five indicator species remained the same (OTU_9, OTU_10, OTU_1, and OTU_13), with the fifth being OTU_4 (a member of Verrucomicrobiaceae). Again, the Enterobacteriaceae members and *S. faecium* were more common across the preservation treatments (1 month, 6 months, and 9 months), and the Spingobacteriaceae and Verrucomicrobiaceae were more common in the unpreserved treatments (starved and fresh).

We also ran a multilevel pattern analysis (multipatt) to determine which bacterial species can be used as indicators of certain treatment groups. When looking at year, there were 26 taxa associated to the year 1920, 6 taxa to 1974, 127 taxa to 1980, 4 taxa to 1982, 16 taxa to 2000, 85 taxa to 2003, 5 taxa to 2004, and 12 taxa to 2018.

Members of Cerasicoccaceae, Chitinophagaceae, *Flavobacterium succinicans*, and another *Flavobacterium* sp. were highly significantly (*p*-value < 0.01) associated with being from 2018. *Bradyrhizobium* sp., *Alicycliphilus* sp., and *Cloacibacterium* sp. were also significant, most strongly associated with coming from 1920 samples. There were five species found exclusively in 1980 samples. No other year had taxa exclusively found in its group samples.

The multilevel pattern analysis for short-term treatment groups showed that no species were specifically associated with snails kept in preservation for 9 months. Starved snails had the highest number of associated taxa at 302, followed by fresh snails at 203, 6-month preservation has 9 associated taxa, and 1-month preservation had 6 associated taxa.

### Microbial richness

The average richness per gut sample was 434.55 ± SD 241.42 bacterial species, with a maximum of 1338 species and a minimum of 42 species.

A negative binomial generalized linear model was used on all museum and field samples from 1920 to 2018. The collection year was not a significant predictor of microbial richness across all samples (*p*-value = 0.35, Table 3). The location was also not significant predictor when controlling for year (Table 3). Palmer drought index was not a significant predictor of microbial richness, apart from snails deriving from “moderately moist” seasons, which had significantly lowered richness (expβ =  − 0.64; *p*-value = 0.05). The elevation was also not a significant predictor of microbial richness (expβ < 0.001; *p*-value = 0.23).

**Fig. 5:**
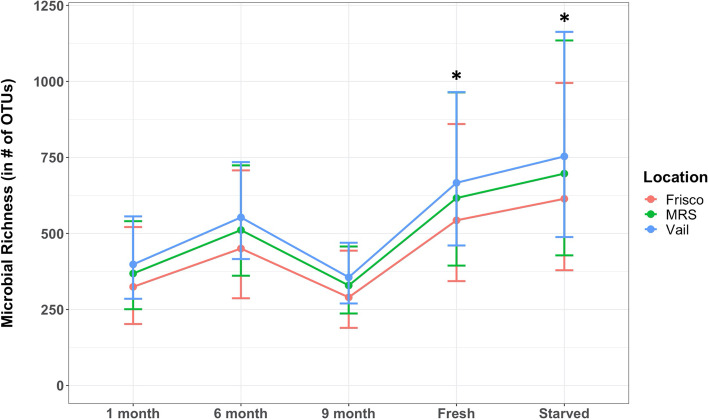
Average microbial richness in varying short-term preservation treatments, differentially across locations. Treatments that underwent no preservation prior to DNA extraction (fresh and starved) had significantly higher richness. ^*^Significant difference. Error bars indicate standard error

A negative binomial generalized linear model was used on all field samples collected during the summer of 2018. For a fixed location, undergoing no preservation (as part of the fresh and starved treatments) was a significant factor in gut microbial richness (Fig. 5).

Undergoing no preservation prior to gut extraction as part of the fresh treatment significantly increased microbial richness (expβ = 0.52; *p*-value = 0.03, Table 4) for a given location, while undergoing the starvation treatment and no preservation also significantly increased richness (expβ = 0.64; *p*-value = 0.01, Fig. 5, Table 4). There were non-significant effects of the other short-term preservation treatments, and there was no significant effect of location (Table 4).

Kruskal–Wallis tests showed that evenness changed non-significantly across most years, except for pairwise differences between 2004 and 1920 (*p*-value < 0.05), 2004 and 1974 (*p*-value < 0.05), and 2018 and 1974 (*p*-value < 0.05). Kruskal–Wallis tests showed that Shannon Indices changed non-significantly across most years, except for Shannon Indices in 2004 and 1974 which showed a significant pairwise difference (*p*-value < 0.001).

## Discussion

In this study, we targeted the gut microbiome of *O. strigosa* that were historically collected (between 1920 and 2004), preserved in ethanol, and stored in museum collections along with newly (2018) field collected *O. strigosa* used for short-term preservation treatments. Our results highlighted that long-term preservation does not significantly impact the makeup of the microbiome, in terms of alpha or beta diversity. Most variation occurs within the initial time frame of preservation, and then, microbiome structure appears stable throughout time. Significant variation in community composition is attributed to factors other than preservation period, including geographic location. Other factors not addressed in this study, such as whether the samples were first fixed in formalin and then moved into ethanol preservation, percentage of ethanol used (e.g., 70% versus 95% ethanol), and temperature-controlled storage, as well as fluctuations like freeze–thaw cycles, may have a greater impact on microbiome makeup than storage time [6, 14, 29].

### The core microbiome

The core microbiome of *O. strigosa* found in this study is consistent with that found in Chalifour & Li [13]. They consisted of two members of family Enterobacteriaceae and one unidentified *Spingomonas* species. These three OTUs made up nearly a third of all sequenced reads. Despite being collected across a broad temporal and geographic gradient within Colorado, our results suggest that there is a shared core microbiome across all these *O. strigosa* specimens. In addition, both historic and 2018 samples shared 3603 OTUs, or around half of all sequenced OTUs, showing that there is stability across the 98-year period of gut microbiome residents.

Members of Enterobacteriaceae, Sphingobacteriaceae, and Sphingomonadaceae have all been associated with other snail species’ gut microbiomes [10, 30, 31]. While associations between *Oreohelix* and their gut bacteria have barely been studied, their diet and habitats suggest that the microbiome may have important functional roles in cellulose breakdown. Among some of the representative *O. strigosa* microbiome species, *Flacobacterium succinicans* has been found in Daphnia guts [32], as well as soil and fresh water. Members of Roseomonas have been found in the gill and gastrointestinal microbiomes of Tilapia [33]. *Stenotrophomonas maltophilia* and other *Stenotrophomonas* species have been identified in bark beetle guts, and *S. maltophilia* in particular has shown cellulolytic activity [34].

### Microbial composition

#### Preservation and gut microbiome community composition

Our results show that environmental factors are more likely to affect the microbiome composition than preservation factors. Long-term preservation seems to impact the makeup of the microbiome only marginally (Fig. 4A) compared with location which explained much more of the microbial composition variability (Fig. 4B) and does not significantly impact the richness of the microbiome (Fig. 5). Only 2.25% of variation in gut bacterial diversity was explained by the year the snail samples were collected (Fig. 4A, *p*-value < 0.001). In contrast, 12.38% of variation in microbiome composition was explained by the location the snail sample was collected (Fig. 4B, *p*-value < 0.001). So, samples from 50 years ago have just as much potential to answer biological questions as those from 10 years ago or less. Importantly, most samples, regardless of being historic or current, passed all quality control steps within the USEARCH pipeline [18], and those that did not meet the minimum read count were from later years (2004 and 2018). This preliminary, but vital step, shows that next-generation sequencing technology can characterize bacterial communities of historic specimens as well as contemporary specimens. In other studies, sequencing platform variability has a negligible effect of microbiome makeup differences across samples; rather, storage conditions are far more important in driving differences [35].

Importantly, we could not gather ethanol-preserved O*. strigosa* from the same location over the entire temporal range. As we were bound by the limitations of what previous collectors in the past century had deposited in the museum, we were forced to sample periodically from varying locations. We did attempt to resample several locations from the historic collections only to find that the historic *O. strigosa* populations no longer exist in those locations in modern times. This is potentially due to human disturbance, changing climatic conditions, or a combination of these two factors. The focus of this study is to deduce the effect of time in long-term preservation on gut microbiome composition; however, we could not ignore the potential effects of location differences and felt it important to include them as possible driving factors of microbiome change.

Similar studies of museum specimens over shorter periods of time have been conducted with varying results. The longest study known to us is one by Neu et al., 2021, which investigated microbiome composition and richness of the ethanol-preserved marine bivalve, *Donax gouldii*, over the span of one decade [2]. As with our study, Neu et al. show that the preserved, historic tissue provided high-quality information about temporal trends across the 11-year period. Importantly, these samples were preserved in 95% ethanol at 4 °C, rather than in 80% ethanol at room temperature. Nonetheless, their result is consistent with our findings that historic samples can be effectively used over longer time scales to study animal microbiomes [2]. In agreement with our study, Neu et al. 2021 found microbiome composition and richness across the temporal range were stable, and there was a core microbiome found across all time points [2]. Another study of a smaller scale by Heindler et al. (2018) showed that preserved fish specimens had a very clear change in microbiome composition over time, but it is due to true biological shifts and not preservative biases [6].

Short-term preservation accounts for more of the variation between 2018 samples than location (Fig. 4C, D), but most changes happen between unpreserved and preserved treatments, rather than across each preservation time point. The location explained more of the variance when compared to the binary explanatory variable of the presence of preservation (whether a sample was preserved at all [1 month, 6 month, 9-month treatments] or never preserved [fresh and starved treatments]). In another short-term study only preserving specimens up to 60 days, the effect of preservation was also detected in PERMANOVAs in explaining microbiome compositions [36]. However, this study also tested different storage mediums (e.g., ethanol, freezing, RNA Later, etc.) and found that ethanol preservation was the most consistent in working well over time to preserve the microbiome and there was no difference between fresh and preserved insect samples; the effect of preservation was more prevalent in the other methodologies like RNALater. As ethanol was the most stable of the methods tested, this could explain why samples preserved long term in ethanol solution showed stability in their composition, and samples preserved for the short-term monthly periods also remained more similar to each other than those not preserved at all.

Variation due to anesthetization method could contribute to the initial shifts of gut microbiome diversity immediately after preservation in this study. We drowned snails to anesthetize them before fixing them in ethanol, as it is the traditional procedure for snail sacrifice. This method may have caused snails to expel some amount of microbial species into the water that is then lost in every treatment downstream of this step, thus explaining why non-preserved treatments were more similar to each other and preserved treatments of any age were also more similar. One study postulates that drowning actually degrades DNA [37]; however, there is considerable benefit in obtaining well-relaxed soft bodies. Well-relaxed bodies make removal from the shell less destructive and allow the body to come out in one piece, which is important for preserving the morphology of the snail and performing accurate dissections [38]. Additionally, a later study by Kruckenhauser et al. showed that drowning did not affect the DNA quality needed for successful PCR amplification and other DNA-based methods [38]. Therefore, if drowning or other relaxation methods (e.g., magnesium chloride) are necessary for preserving certain taxa, it may be valuable to sequence a few freshly collected individuals as a microbiome reference.

Differences between preserved and non-preserved samples could be due to the physiology of some microbes. There may be some microbial taxa that do not preserve well in 80% ethanol and are thus less represented in the preserved samples, also explaining why richness is lower in those short-term samples. However, multiple studies have shown that ethanol preservation is the ideal method for preserving microbiomes [36, 39]. Additionally, although we believe ethanol was the only preservative used in our samples, in some older museum specimens, formalin was initially used to preserve specimens, starting in 1891 and gaining popularity into the first quarter of the twentieth century [40]. Formalin can cause crosslinking among DNA molecules, even resulting in depolymerization of the DNA [6, 41, 42]. Unfortunately, the initial preservative is not always documented in museum records. To allow future researchers to use museum samples for microbiome study, detailed descriptions of sampling and preservation procedures should be kept to reduce variability from preservation practices in samples.

### Location and gut microbiome community composition

Significant results from this study substantiate the idea that snail gut microbiome changes are partially driven by geographic distances. When controlling for year, the location was a significant predictor of gut microbiome composition and explained 12.38% of variability between all samples and 16.91% of variability in just 2018 samples.

In other systems, the location also appears to account for significant differences in microbiome variation. Generally, the geographic location has been shown in many sponges to drive intraspecific microbiome variation [43–46]. In another invertebrate, corals, both the microbiome and the pathobiome (pathogenic microbial assemblage) are dependent on geographic location [47]. Both honeybees and oysters show gut communities that differ based on sites, and in bees, among colonies within sites [48, 49].

Although geographic location appears to be a significant driver of a large proportion of the variation across samples, it is possible that this pattern is driven by other environmental factors that co-vary with location, such as habitat, temperature, drought level, or diet. For example, animal diets have been shown to be strongly correlated with the bacterial compositions present in their gut microbiomes [3]. Members of the same host species originating from different habitats become exposed to distinct microbial communities and are then colonized by distinct gut microbial communities [12]. Shifts in diet happen regularly for wild animals, based on food availability, season, etc., and are strongly correlated with habitat and location [12]. For example, Black howler monkeys show distinct differences in gut community composition based on four habitats, each unique in what diets it enables [12]. Diet has a significant effect on other animals’ microbiomes, like wild lizards [50] and sea urchins [51]. This may also be common in snails, as in various other invertebrate host/bacterial symbiont systems, microbes are transmitted horizontally [52]. Many land snails use a generalist feeding strategy and thus have evolved unique gut microbiomes to efficiently breakdown and use a variety of tough, cellulolytic, vegetative materials for their own nutrition and growth [10, 53]. Some terrestrial snails are known to augment their gut microbiome through horizontal transmission or the collection of environmental bacteria from their surrounding habitat through eating [10]. Another major factor that may be at play due to horizontal transmission is the geology of the populations’ location. As a member of a calciphilous family, *Oreohelix* has already shown phenotypic plasticity in its shell ornamentation due to geologic factors like the availability of calcium carbonate [54]. Local adaptations to geology may also be present in the gut microbiome, especially if *Oreohelix* uptake bacteria directly from soil or rocky substrates of their habitats. Thus, it is likely that each *O. strigosa* snail population harbors a gut microbiome that is unique to their specific habitat and thus a geographically specific diet, regardless of how long it has been in preservation for.

Palmer drought index and elevation as explanatory variables did not significantly drive any changes in microbial composition. In other animals, drought can be a good indicator of resource abundance or restriction. In buffalo, shifts in gut microbiome composition and the enterotypes present are driven by resource restriction associated with drought conditions, also resulting in decreased number of microbial species present in the gut [55]. However, *O. strigosa* microbiomes do not show significant disturbances due to drought. This may be because these snails are more drought tolerant than other animal species; they regularly aestivate when conditions are too hot or dry, and so their microbiome may be more acclimated to shifts in habitat favorability [56]. In general, many snails are known to exist synergistically with drought, altering their feeding apparatus in unison with drought events [57] and have a high tolerance and survival rate to prolonged drying events [58].

While not specifically investigated in this study, forms of anthropogenic disturbance can also drive changes in microbiome composition in other animal species [59, 60]. The Front Range of Colorado—where our snail samples originated from—has seen many striking changes in the past century, including expansion of residential and commercial land development marked by intensive land-use conversions, even in rural, mountainous areas [61]. As *O. strigosa* are typically ecologically specialized to small segments of montane ecosystems [62], disruptions in the form of quarrying, habitat fragmentation, and general human presence may contribute to biodiversity loss and greatly impact their microbiomes or cause dysbiosis.

### Indicator species analysis

The indicator species present in varying treatment groups of *O. strigosa* are all common across other gastropod microbiomes. Members of Verrucomicrobia are found in planorbid snails, including *Bulinus africanus* and *Helisoma duryi* [63]. Members of Spirosoma have been characterized in the Hawaiian tree snail *Achatinella mustelina* [64]. Enterobacteriaceae are present in the land snails *Achatina fulica*,* Cornu aspersum*,* Helix pomatia*, and *Helix aspersa* and are hypothesized to take part in food fermentation in the gut [10, 31, 65, 66].

In general, the later years sampled (from 2000 through 2018) showed Palmer drought indexes of extreme or severe drought, while most moister years occurred in the 1980s and before. In these later years, the family Enterobacteriaceae are more prevalent than in earlier years (Fig. 3A). Members of Enterobacteriaceae have been found to have a significant seed germination-promoting effect and stimulate seedling growth in plant species inoculated with these strains under severe drought stress [67]. As *Enterobacter* strains are drought tolerant and have a diverse growth range under stressful conditions, they may be more common in snail hosts that are also experiencing drought stress [68]. Snails may be feeding on plant matter that was assisted in growing by Enterobacteriaceae that are still present in the plant, thus being exogenously passed to the snail gut. Likewise, a member of family Cerasicoccaceae was associated to 2018 snails, which has also been associated with drastic environmental changes (such as changes in precipitation) in planktonic bacterial communities [31]. While there are shifts in indicator species for particular years, the overall microbial community compositions do not differ significantly among the years, explaining why drought index is not a significant driver. Similar results have been found in other systems. For example, thermally stressed barrel sponges show no significant community changes of the microbiome compared with non-stressed sponges [69]. There was however evidence of functional changes between these groups, with stressed sponges having lowered abundance of microbial photosynthetic proteins [69]. While the overall snail gut microbiome composition may stay similar throughout moist and drought years, there may be some key microbial players that do change in abundance in accordance with climate-induced shifts. Therefore, future studies should focus on understanding how ecological factors impact the abundance of different functional microbial groups, in addition to overall microbiome compositions.

### Microbial richness

Microbial richness shows a low amount of variation driven specifically by year. Consequently, community composition, rather than variation in richness, is responsible for the observed variation in the microbial communities across these snails. Elevation showed no significant effect on richness, and neither did Palmer drought index, apart from a significantly lowered richness in snails collected in moderately moist seasons. Overall, our findings support that long-term preservation allows for unbiased quantification of the microbial richness of the gut microbiome, as it remained stable over the 98-year temporal range of this study.

We found that the initial transition from being non-preserved to preserved had the most impact on altering the richness of the microbiome. For the 2018 samples, significant differences lie between the completely unpreserved treatments (fresh, starved) and the treatments with some level of preservation (1 month, 6 months, 9 months). For a fixed location, undergoing no preservation (as part of the fresh or starved treatments) was a significant factor in increasing gut microbial richness. This may be due to the method of fixation and preservation used (e.g., drowning method, ethanol vs. formalin) as discussed in the microbial composition discussion above.

Nonsignificant differences between the richness of different communities could be due to host related factors. Varying diet composition, as discussed before, could explain the greater variation in richness seen when looking specifically at location. The stage of development of the host can also create variations in microbiome richness [13, 70]. As all the snails used in this study were adults, we do not think development stage was a factor here; however, it should be considered in future studies of this nature. Host gut length has been suggested as a factor in shaping the microbiome and could also be considered in future studies [70]. Additionally, in other studies, host genotype can be extremely important in shaping the gut microbiome and should be considered when using museum specimens from multiple species [11, 71].

## Conclusions

The use of advanced molecular technology offers a novel method to study microbial communities of ethanol-preserved museum specimens. In this study, we used next-gen high-throughput sequencing technique to investigate the bacterial diversity of museum specimens of the snail species *O. strigosa* across nearly a century of preservation age*.* We have demonstrated that specimens from all ages generated high-quality sequencing data, and the snails’ core microbiomes were stable across all museum specimens. Both long- and short-term preservation time explained little variation in the data, with other factors like location explaining more of the variation between samples. Further research is required to better characterize other potential driving factors of microbiome variability in *O. strigosa*, which include initial preservation and storage methodologies, especially for museum samples.

In the future, similar studies need to be done on more diverse museum specimens and on various types of microbiomes (mucosal, fecal, whole body, etc.). Consistent methods of preservations are necessary to definitively reduce sources of variability across microbiome sample compositions. At least, consistent records of preservation techniques along with detailed locality notes are helpful for researchers conducting microbial ecology studies. It is similarly vital for ecologists to communicate closely with museum workers to create a coordinated approach for sample lending, destruction, and the return of usable DNA to the museum institution [72]. This study should encourage further use of museums as a resource for studying microbial ecology across time and space.

## Supplementary Information

**Additional file 1:** **Supplementary Table 1**. Chalifour, Elder, & Li 2021—Gut microbiome of century-old snail specimens stable across time in preservation.

## Data Availability

The data that support the findings of this study are openly available in FigShare at 10.6084/m9.figshare.19944563.v1 and 10.6084/m9.figshare.19944656.v1. Related metadata can be found at (10.6084/m9.figshare.19944563.v1) and Supplementary Table 1 provides unique sample identifier tags that can be matched to both the deposited sequence data (10.6084/m9.figshare.19944656.v1) and deposited metadata (10.6084/m9.figshare.19944563.v1), along with all associated museum database information.

## Abbreviations

AIC: Akaike information criterion
ANOVA: Analysis of variance
CUMNH: University of Colorado Museum of Natural History
DNA: Deoxyribonucleic acid
MRS: University of Colorado Mountain Research Station
Multipatt: Multilevel analysis of pattern
NMDS: Non-metric multi-dimensional scaling
NOAA: National Oceanic and Atmospheric Administration
OTU: Operational taxonomic unit
PCR: Polymerase chain reaction
PERMANOVA: Permutational analysis of variance
rRNA: Ribosomal ribonucleic acid
SIMPER: Similarity percentage
SD: Standard deviation
USGS: United States Geological Survey
